# Which Risk Factors Matter More for Psychological Distress during the COVID-19 Pandemic? An Application Approach of Gradient Boosting Decision Trees

**DOI:** 10.3390/ijerph18115879

**Published:** 2021-05-30

**Authors:** Yiyi Chen, Ye Liu

**Affiliations:** 1 School of Geography and Planning, Sun Yat-Sen University, Guangzhou 510275, China; 2 Guangdong Key Laboratory for Urbanization and Geo-Simulation, Sun Yat-Sen University, Guangzhou 510275, China; lwish@outlook.com

**Keywords:** coronavirus disease 2019 (COVID-19), psychological distress, Kessler psychological distress scale, relative importance, machine learning approach

## Abstract

Background: A growing body of scientific literature indicates that risk factors for COVID-19 contribute to a high level of psychological distress. However, there is no consensus on which factors contribute more to predicting psychological health. Objectives: The present study quantifies the importance of related risk factors on the level of psychological distress and further explores the threshold effect of each rick factor on the level of psychological distress. Both subjective and objective measures of risk factors are considered in the model. Methods: We sampled 937 individual items of data obtained from an online questionnaire between 20 January and 13 February 2020 in China. Objective risk factors were measured in terms of direct distance from respondents’ housing to the nearest COVID-19 hospital, direct distance from respondents’ housing to the nearest park, and the air quality index (AQI). Perceived risk factors were measured in regard to perceived distance to the nearest COVID-19 hospital, perceived air quality, and perceived environmental quality. Psychological distress was measured with the Kessler psychological distress scale K6 score. The following health risk factors and sociodemographic factors were considered: self-rated health level, physical health status, physical activity, current smoker or drinker, age, gender, marital status, educational attainment level, residence location, and household income level. A gradient boosting decision tree (GBDT) was used to analyse the data. Results: Health risk factors were the greatest contributors to predicting the level of psychological distress, with a relative importance of 42.32% among all influential factors. Objective risk factors had a stronger predictive power than perceived risk factors (23.49% vs. 16.26%). Furthermore, it was found that there was a dramatic rise in the moderate level of psychological distress regarding the threshold of AQI between 40 and 50, and 110 and 130, respectively. Gender-sensitive analysis revealed that women and men responded differently to psychological distress based on different risk factors. Conclusion: We found evidence that perceived indoor air quality played a more important role in predicting psychological distress compared to ambient air pollution during the COVID-19 pandemic.

## 1. Introduction

The 2019 coronavirus (COVID-19) pandemic is a health threat that has spread throughout the world [1,2,3]. Patients with COVID-19 can suffer from severe pneumonia, pulmonary oedema, or acute renal injury and eventually die of multiple organ failure. As of January 2021, over one hundred million cases of coronavirus have been registered, and more than two million people have died from this virus worldwide. Although different countries have used lockdown measures and vaccine recommendations to control the spread of the virus, the direct effects of the COVID-19 risk factors on people’s mental health should not be overlooked.

Correspondingly, a variety of studies have investigated the direct effects of COVID-19 on people’s mental health outcomes among the general population, e.g., the association between COVID-19-related factors and levels of depression [2,4,5], anxiety [6,7,8], and psychological distress and stress [9,10,11,12,13]. Other studies have evaluated such an association but have focused on health professionals [12,14,15,16,17], college students [6,18,19], and patients [20,21,22]. While these studies provided a substantial evidence base showing that COVID-19-related risk factors are significantly associated with psychological status, to the best of our knowledge, no previous study has identified which related risk factors have the greatest impact on the level of psychological distress.

General studies on human health note that air pollution is a significant risk factor [23]. Previous studies have paid more attention to the association between air pollution and physical health outcomes, such as various adverse respiratory and cardiovascular diseases [24], while recent epidemiological studies have indicated a feasible correlation between air pollution and psychological health. Specifically, a growing number of studies have examined the association between air pollutants and a person’s depression and psychological distress. Studies conducted in the US, Canada, and China provide most of the literature on this topic though the results have been mixed. One study conducted in the US found no association between the short-term air pollutant level and the depressive symptoms of participants (age over 65), while two other studies reported positive relationships between pollution effects and depression and anxiety symptoms [25,26]. A longitudinal study conducted in the US confirmed that PM2.5 was significantly associated with increased psychological distress [27]. One Canadian study found a positive relationship between ambient air pollution (PM2.5 and NO) and psychological distress through using the measure of the Kessler Psychological Distress Scale (K10) [28]. Finally, a recent study in China indicated that an increase in the previous week’s PM2.5 contributed to an increase in the prevalence of depression, and such effects were more pronounced in the spring and summer and in eastern and southern areas [29]. The findings from these works are promising but not conclusive, since many of these studies emphasized only linear or non-linear associations, or they utilized inconsistent measures and methodologies [30].

In addition, there are other influences. Recent studies have pointed out the importance of perceived factors on psychological health status. In 2008, the Blacksmith Institute World’s Worst Polluted Places report listed indoor air pollution as one of the world’s worst toxic pollution problems [31]. Indeed, evidence has demonstrated that indoor air quality plays an essential role in determining people’s health, since people in modern society spend over 65% of their time in their own residence [32]. Research has found that indoor air quality within buildings and homes might be worse than outdoor air quality, even in industrial cities [33]. However, the findings of previous studies investigating the relationship between indoor air quality and health symptoms are mixed. For example, one study found that the perceived indoor environmental quality was associated with psychological distress with respect to the workplace by using a seven-point Likert scale: 1 (unsatisfactory) to 7 (satisfactory) [34]. Another study in China examined the association between living environment and self-reported health, with a special focus on the differences between rural and urban residents in relation to environmental health [35]. However, they found no significant association between exposure to self-reported air pollution and self-rated health. Limited studies have evaluated the direct effect of self-reported or perceived air quality on psychological health benefits, especially during the COVID-19 outbreak, when respondents in the majority of Chinese cities were required to stay in their residence and were allowed to venture out only to obtain necessary medical help or to purchase daily food. Prolonged quarantine restrictions increase the time people spend indoors, so a lower level of self-reported indoor air quality might exacerbate the level of psychological distress.

Additionally, studies have indicated that risk factors related to sociodemographic characteristics and health are associated with symptoms of psychological distress. For example, studies reported that females were more likely to develop symptoms than their male counterparts [12,36,37]. Younger participants were more likely to perceive a higher level of psychological distress compared to the elderly [13,37]; participants who had a chronic disease and reported a lower self-rated health level tended to have a higher probability of suffering from depressive symptoms compared to those who did not [12,38]; and participants who were current smokers or who had a current specific level of alcohol consumption were associated with a higher level of psychological distress [39], while participants who took part in moderate or sufficient physical activity reported less psychological distress [40]. Nevertheless, these studies quantified the significant associations between sociodemographic characteristics and psychological distress, and thus further investigation is required to identify which sociodemographic characteristics have a greater influence on psychological distress.

The novelty of this study is that it examines how different risk factors may affect psychological distress by applying gradient-boosting decision trees (GBDT) to national web-based data on Chinese residents during the early stage of the COVID-19 outbreak, emphasizing the threshold effects of both objective and perceived risk factors on the level of psychological distress. This study aims to address the following research questions: (1) how important is the effect of objective and perceived risk factors in predicting the level of psychological distress, (2) which risk factors most affect the level of psychological distress, and (3) do different risk factors have threshold effects in predicting the level of psychological distress? Uncovering these differences in the importance of risk factors should help to advance epidemiological research in this area.

## 2. Materials and Methods

### 2.1. Method

The GBDT was applied in this study to serve as a new machine-learning approach in the field of urban planning and development. By using this approach, recent studies have investigated the relationship between the built environment and travel behavior [41], and population density and waist-hip ratio [42]. Compared to the traditional regression model, GBDT has several advantages. Firstly, GBDT can efficiently handle complex and non-linear correlations while maintaining a relatively high prediction accuracy [41,42]. One reason is that the GBDT approach uses decision trees to classify predictors and estimate the outcome by minimizing the loss function. As noted in the study, “Models are fit by minimizing a loss function averaged over the training data, such as the squared-error or a likelihood-based loss function” [43]. Second, the GBDT can effectively handle missing data through marking such data with comprising information rather than missing it at random, so the missing value is treated as a new category during the tree building. Lastly, the GBDT can compute and rank the relative importance among predictors contributing to the response variable, whereas traditional statistical models find this difficult to achieve [44].

The equation is derived from the research [43] and is summarized as follows:

Initialize

(1) $$f_{0}(x)=\arg\min_{\;\gamma }\sum {}_{i=1}^{N}L(y_{i},\gamma )$$

For *m* = 1 to *M*:

For *I* = 1, 2, …, *N* computer

(2) $$r_{\min}=-{\left[\frac{\partial L(y_{i},f(x_{i}))}{\partial f(x_{i})}\right]}_{f=f_{m-1}}$$

Fit the regression tree to the targets $r_{im}$ giving terminal regions

*R_jm_*, *j* = 1, 2, …, *J_m_* (3)

For *j* = 1, 2, …, $J_{m}$ compute

(4) $$\gamma_{\min}=\arg\underset{\gamma }{\min}\sum_{x_{i}\in R_{jm}}L(y_{i},f_{m-1}(x_{i})+\gamma )$$

Update

(5) $$f_{m}(x)=f_{m-1}(x)+\sum {}_{j=1}^{Jm}\gamma_{jm}I(x\in R_{jm})$$

Output

(6) f^(x)=fM(x)=f0(x)+∑m=1M∑j=1JcjmI(x∈Rjm)

Three steps are conducted to generate the equation. Firstly, the approach initializes the optional constant model to minimize the loss function L(y,f(x)). Then, in the second step, the equation is constructed by four sub-steps at each interaction m. The negative gradient of the loss function is calculated as an estimate of a generalized or pseudo residual (Equation (2)). It further fits the regression tree to the target. It is important to note that two layers of loops are nested in the algorithm, namely, the number of iteration rounds m and the sample i. The regression tree of rounds m is obtained through applying the calculated formula ($x_{i},r_{ti}$) to fit into a regression tree of CART. In terms of sub-step (Equation (3)), J represents the size of each of the constituent trees. Then, the equation calculates the optimal fitting value $\gamma_{\min}$ for the leaf area (loss function minimization) and updates the strong learner. Lastly, it reports the results of the final model. 

GBDTs build models stagewise and update models by minimizing a loss function’s expected value. A shrinkage strategy is applied by the GBDT to prevent over-fitting and improve prediction accuracy [45,46]. However, an overfitting problem might exist when training data are fitted too closely. Therefore, three parameters are introduced to prevent the over-fitting problem and promote prediction accuracy, namely, the number of trees (M), learning rate (ξ), and tree complexity (C). It should be noted that the tree complexity determines the model complexity and how well the model fits, while the learning rate (shrinkage) is calculated to scale the contribution of each base tree model by introducing a factor of ξ (0 < ξ ≤ 1) as shown below:

(7) $$f_{m}(x)=f_{m-1}(x)+\xi \times \beta_{m}\sum \frac{J}{j=1}\gamma_{jm}I(x\in R_{jm}),\;where\;0<\xi \leq 1$$

The smaller ξ is, the greater the shrinkage becomes, which indicates that the over-fitting problem can be solved by reducing or shrinking the impact of each tree. However, it is worth noting that a large number of trees might be added to the model during the process. Tree complexity C, which denotes the number of splits (or the number of nodes), is calculated for fitting each decision tree. It represents the depth of variable interaction in a tree. In accordance with the research [43], 2 ≤ C ≤ 5 indicates that the model generally works well. Optional performance of the model depends on choosing the combination of the number of trees (M), learning rate (ξ), and tree complexity (C).

Generally, predictors are rarely equally relevant, as they have different influences on the response variables during the data mining process. Thus, it is necessary to learn the relative importance or contribution of each input predictor in estimating the response. Compared to the traditional modelling approach, the GBDT method can systematically identify and rank the influences of predictors on response predictions. 

For a single decision tree *T*, in accordance with the research [47], the equation can be written as follows:

(8) $$I_{\kappa }^{2}(T)=\sum_{t=1}^{J-1}{\overset{\wedge }{\tau }}_{t}^{2}I(v(t)=\kappa )$$

where the summation applies to the non-terminal nodes *t* of J-terminal node tree *T*, $x_{\kappa }$ denotes the splitting variable correlated with node *t*, and ${\overset{\wedge }{\tau }}_{t}^{2}$ denotes the corresponding improvement in a square error as when conducting the splitting variables $x_{\kappa }$ as the non-terminal node *t*.

### 2.2. Study Area and Data Description

The data were collected using an online questionnaire between 20 January and 13 February—that is, during the early stage of the outbreak of COVID-19 in China. Using the crowdsourcing platform ‘Wenjuanxing’, 1037 responses were gathered from the online questionnaire. Following the data cleaning process, 100 samples were dropped from the study because respondents had provided invalid information in response to other questions relating to the quarantine location. This left a total of 937 responses that could be used for the statistical analysis. The survey explored individuals’ demographic characteristics, health risk factors, psychological distress, and perceived risk factors. The study spanned 230 cities in China. The study received ethical approval from the School of Energy, Geoscience, Infrastructure and Society, at Heriot-Watt University.

### 2.3. Variables Definition

#### 2.3.1. Response Variable

The response variable in this study is psychological distress. Psychological distress was assessed with the Kessler Psychological Distress Scale K6 score [48]. It is acceptable to use K6 to measure people’s distress level, since it has been widely used and has evidenced reliability and validity across a wide variety of mental health surveys [49,50]. The scale includes six items associated with psychological distress in the previous 4 weeks. It is based on six questions, which were used in our survey as follows: How often have you been feeling (a) nervous, (b) restless or fidgety, (c) so sad nothing could cheer you up, (d) hopeless, (e) everything was an effort, and (f) worthless? The answer to each question is given via a 5-point Likert scale ranging from 0 ‘never’ to 4 ‘very often’. A K6 result of 13 or above indicates high psychological distress, a score of between 8 and 12 denotes moderate psychological distress, and a score of between 0 and 7 denotes low psychological distress [48].

#### 2.3.2. Objective Predictors

In this study, objective predictors included three key risk factors that were derived from recent research: direct distance from the respondent’s housing to the nearest COVID-19 hospital [10], direct distance from the respondent’s housing to the nearest park [51], and the air quality index (AQI) [52]. The data regarding the parks were obtained from Beijing City Lab (Beijing City Lab, 2019, Data 40, Urban green lands in main Chinese cities 2017, http://www.beijingcitylab.com (accessed on 30 May 2021)) which shared information on 16,721 urban green spaces in 287 Chinese cities in 2017. The data package included the size of different parks, the landscape shape index, and the geocoordinate, which made it possible to conduct the spatial analysis. Data regarding distance to the nearest hospital were obtained from the website (http://file.caixin.com/datanews_mobile/interactive/2020/fever accessed on 9 February 2020), which recorded the location (including the geocoordinate) of hospitals that were specifically used for curing COVID-19 patients. Regarding the AQI, data were acquired from a real-time remote inquiry website (Airborne Fine Particulate Matter and Air Quality Index. Secondary Airborne Fine Particulate matter and Air Quality Index 2020. Available online: http://www.pm25.in/ (accessed on 7 July 2020)) that provides an hourly value for the AQI with real-time concentration and the 24 h moving average of air pollutant indicators, such as PM2.5, PM10, SO_2_, CO, NO_2_, and O_3_. AQI observation data from 20 January to 13 February were collected and cleaned and then connected to the survey data through the ‘Near’ id. The ‘Near’ id was generated through the ‘Near’ tool. In this study, the spatial ‘Near’ tool in ArcGIS was used to calculate the direct distance from each property to the nearest COVID-19 hospital and the nearest park. We also measured the direct distance from each respondent’s location to the nearest monitoring stations that provided the AQI.

#### 2.3.3. Perceived Predictors

Perceived predictors are defined by a limited number of characteristics applicable to the Chinese context, including perceived distance to the nearest COVID-19 hospital, perceived indoor air quality, and perceived environmental quality [10]; these were measured on 5-point Likert scales. Perceived distance to the COVID-19 hospital was measured by the following question: ‘How would you rate the distance from your house to the nearest COVID-19 hospital?’, which referred to a hospital specifically used for curing COVID-19 patients. The options were (1) very far, at least an hour’s drive; (2) far, at least half hour’s drive; (3) close, at least 10 to 30 min’ drive; and (4) very close, a 5-min drive. Perceived air quality was assessed with the following question: ‘How would you rate your indoor air quality level overall?’ Response categories ranged from (1) extremely bad to (5) extremely good. Perceived neighborhood environmental quality was measured by the following question: ‘How would you rate your neighborhood environment overall?’ Response categories ranged from (1) neighborhood environment maintained in very poor quality to (5) neighborhood environment maintained in very good quality.

#### 2.3.4. Health and Sociodemographic Predictors

Many studies have identified several lifestyle variables as significant predictors of psychological distress; physical health status, physical activity, and behaviors such as smoking and drinking are significantly associated with psychological distress [53,54,55,56,57,58]. Accordingly, in this study, we investigated five potential health risk factors that may affect the response predictors: self-rated health level, physical health status, physical activity, and current smoker or drinker. Respondents’ self-rated health was measured with the question: ‘In general, how would you rate your general health status?’ with possible choices ranging from (1) very poor to (5) very good. Respondents’ physical health status was measured with the question: ‘Have you been diagnosed with a chronic disease in the past six months?’ This item was then coded (1) yes or (0) no. Respondents’ regular physical exercise was assessed with the question: ‘How often have you exercised during the outbreak of COVID-19?’ Response categories ranged from (1) never to (5) very often. Regarding behaviors, we measured smoking and drinking with two items. Smoking was measured with the following question: ‘Have you smoked in the last month?’ This item was coded (1) for smokers or (0) otherwise. Similarly, we measured drinking by the following question: ‘Have you drunk alcohol more than 3 times per week in the last month?’ This item was coded (1) for drinkers or (0) otherwise. Additionally, we controlled for sociodemographic predictors, such as age, gender, marital status, educational attainment level, and household income level.

### 2.4. Reliability and Validity

Before running the GBDT model, the variables were first analyzed in SPSS (IBM, Armonk, NY, USA) to test their reliability. Cronbach’s alpha is the coefficient used to estimate the reliability of instruments based on internal consistency [59]. Cronbach’s alpha reliability coefficient normally has values from 0 to 1, where a higher value refers to a greater internal consistency of variables in the scale. The results show an acceptable validity of the K6 with a reliability coefficient (alpha) of 0.73 > 0.70. A reliability coefficient (alpha) of 0.70 or higher is considered an acceptable reliability in previous studies in SPSS [60,61,62].

## 3. Results

We ran the “gbm” package in R to apply the GBDT model used by the study [63]. Five-fold cross-validation was applied to minimize the estimate and overfitting errors. We conducted Gaussian regression to reduce the squared error. In this study, we set the maximum of 10,000 trees, kept the learning rate at 0.001, the interaction depth at 5, the bag fraction at 0.5, and the training fraction at 0.5. After 2286 boosting interactions, the model generated the best results.

Table 1 defines and describes the variables used in this study. Table 1 presents the descriptive characteristics for the sample (*n* = 937). The respondents were predominantly young, with 59% aged between 18 and 34 years, and 34.7% were male. In addition, 60.8% of the respondents were married, 24.5% of the respondents had attained a degree and above, and 35.5% of the respondents earned over 10,000 yuan monthly, which was higher than the national average of 4340 yuan according to the 2019 wave of the China Household Finance Survey (CHFS) (CHFS has conducted several follow-up surveys since 2011). Regarding respondents’ lifestyle characteristics, 21.3% of the respondents reported that they slept less than 7 h per day, and 24.6% reported having slept over 9 h per day. Nearly half of the respondents lived a sedentary lifestyle, exercising only once a week or less. Additionally, 21.7% of the respondents reported having smoked in the previous four weeks, which was lower than the national average of 26.8% reported by the fourth wave of the CFPS conducted in 2018 (the China Family Panel Studies (CFPS), launched by Peking University, is a longitudinal social survey consisting of four waves thus far. The four waves of the CFPS include CFPS 2012, CFPS 2014, CFPS 2016 and CFPS 2018. CFPS 2010 was conducted as the baseline survey). Furthermore, 20.1% of the respondents reported having consumed alcohol more than three times per week in the previous month, which is higher than the national average of 14.0% reported by the fourth wave of the CFPS conducted in 2018. Less than 50% of the respondents reported being satisfied with their neighborly relationships, while about 38% of the respondents perceived that their environment was maintained in good and very good quality. Furthermore, less than 15.5% of the respondents rated their indoor air quality as bad or extremely bad. Of note, the average distance from residence to park was 37.8 km, and the average distance from residence to the nearest COVID-19 hospital was 67.1 km, whereas 21.9% of the respondents reported living a very far distance from a COVID-19 hospital. Overall, the sample primarily contains young, relatively well-educated adults; this perhaps explains the average K6 score of 9.2, which suggests that the respondents perceived only moderate psychological distress. In comparison, the average K6 score reported in the CFPS 2018 rating was 9.4, which is higher than the score of K6 in our study.

### 3.1. Relatively Importance of Predictors

Table 2 shows the relative importance of all independent variables for predicting respondents’ psychological distress. All independent variables were ranked in accordance with the size of their relative importance. The results from Table 2 show that health predictors make a significant contribution in predicting psychological distress level, accounting for 42.32%. Next are objective predictors (accounting for 23.49%), sociodemographic predictors (accounting for 17.91%), and perceived indicators (accounting for 16.26%).

Regarding the health predictors, two essential predictors among all the independent variables are disease and self-rated health, with 17.46% and 16.23% of the predictive power, respectively. One underlying mechanism is respondents who had a chronic disease and a low level of self-rated health, as they were more likely to have a higher level of psychological distress. Being a current drinker and smoker were the third and fourth most important predictors among health characteristics, which contribute to the predicting power with 3.45% and 3.14%, respectively. Physical exercise was ranked last, and accounted for only 2.04%. The most essential predictor among the objective predictors is the distance to parks, which accounted for 9.38%. This is followed by the distance to a COVID-19 hospital (accounting for 7.47%), and AQI ranked third (accounting for 6.64%). Furthermore, neighborly relationships had an important influence on psychological distress, with a relative importance of 6.96%. Perceived indoor air quality played an essential role in affecting respondents’ psychological distress level, with a relative importance of 11.62%. This result is plausible since previous research suggests that residents who have a lower level of perceived indoor air quality are more likely to perceive a higher level of psychological distress.

### 3.2. Association between High-Ranking Predictors and Psychological Distress

**Figure 1:**
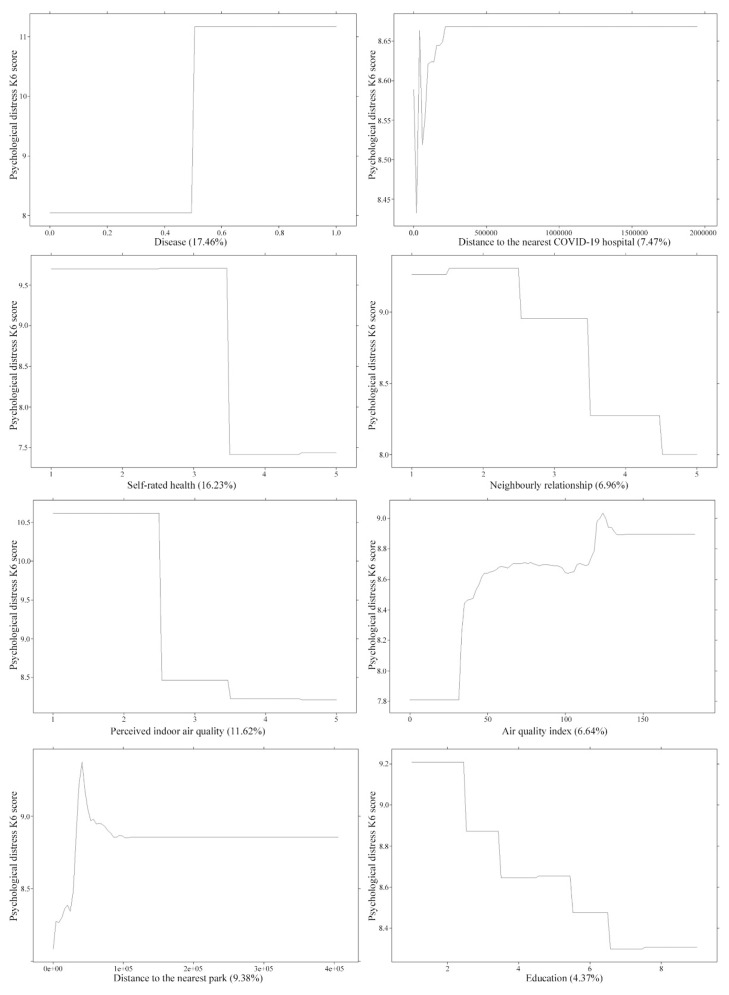
Association between predictors and psychological distress K6 scores.

The results from Figure 1 suggest that respondents who had a chronic disease and low self-rated health level were more likely to perceive a higher level of psychological distress. Furthermore, a higher level of perceived indoor air quality contributed to a lower level of psychological distress. The association between distance to parks and psychological distress was an inverse V-shaped curve. There was a dramatic increase in the level of psychological distress as the distance from residence to parks increased in terms of respondents living adjacent to a green space. Eventually, the level of psychological distress tended to be stable when the distance from residence to parks reached a certain distance. This indicates that respondents’ psychological distress level is sensitive to the distance from their residence to parks, and such sensitivity seems to be mitigated as the distance increases to a certain value. Similarly, we observed a continuous V-shaped curve effect of distance to a COVID-19 hospital on psychological distress. Eventually, it reached a moderate level of psychological distress as the distance from the COVID-19 hospital approached a certain value, which accounts for a score of approximately 9. Moreover, we found that the psychological distress level increased rapidly with respect to an AQI ranging between 40 and 50. The trend levelled off when the AQI was between 50 and 100. Once the AQI exceeded 100, the number of respondents suffering from psychological distress spiked dramatically and then decreased and remained stable for a long time. Regarding the sociodemographic predictors, the results suggest that respondents who had a higher educational attainment level and a good relationship with neighbors were more likely to perceive a lower level of psychological distress. Such effects showed a downward gradient.

### 3.3. Gender Senstive Analysis

Table 3 further displays the gender-sensitive analysis of independent variables on predicting respondents’ psychological distress. Similar to Table 2, all independent variables were ranked in accordance with the size of their relative importance.

For women, the results show that both health predictors and objective predictors make a considerable contribution to predicting the psychological distress level, accounting for 32.05% and 32.01%, respectively. Next are perceived predictors (accounting for 21.13%) and sociodemographic predictors (accounting for 14.80%). In contrast, it is worth noting that there is a slight change in the relative importance ranking among men. The results show that health predictors make a considerable contribution to predicting psychological distress level, accounting for 31.31%. Next are sociodemographic predictors (accounting for 27.57%), objective predictors (accounting for 25.10%), and perceived indicators (accounting for 18.93%).

Regarding the health predictors, we found that disease, which accounted for 16.87%, played a crucial role in influencing psychological distress in men, while self-rated health, which accounted for 25.20%, had the greatest influence on psychological distress among women. The effect of smoking on psychological distress was more pronounced in men (accounting for 1.56%) compared to women (accounting for 0.57%). In contrast, the influence of drinking on psychological distress was more pronounced in women (accounting for 0.80%) compared to men (accounting for 0.63%). In terms of objective predictors, we found that men were more sensitive to the impact of AQI on psychological distress (accounting for 9.74%) than women (accounting for 8.98%), while women were more sensitive to the influence of the direct distance to the nearest COVID-19 hospital on psychological distress (accounting for 14.21%) compared to men (accounting for 8.89%). Regarding the sociodemographic predictors, interestingly, we found that neighborly relationship (accounting for 16.7%) played an important role in influencing psychological distress in men but not in women (accounting for 2.43%). Lastly, in terms of perceived predictors, the results show that perceived indoor air quality (accounting for 13.67%) played an essential role in affecting women’s psychological distress, while perceived environment had a greater effect on men’s psychological distress (accounting for 8.84%). 

## 4. Discussion

### 4.1. Main Findings

Numerous studies have examined the association between different risk factors and mental health during the COVID-19 pandemic and have produced mixed results depending on the different cultural contexts. Some studies have focused on evaluating the effects of the COVID-19 risk factor on psychological health while others have focused more on how sociodemographic characteristics affect psychological health. Yet, it remains unknown as to which factor has a substantial impact on the psychological health level. The present study fills this gap by exploring the varied importance of different risk factors in predicting the level of psychological distress, and it further examines the threshold effect of each risk factor for affecting the level of psychological distress. We found evidence that health risk factors made the greatest contribution to predicting the level of psychological distress. Meanwhile, objective risk factors had a stronger predictive power than perceived risk factors, whereas perceived indoor air quality played a more important role in predicting psychological distress compared to the ambient air pollution during the COVID-19 pandemic.

### 4.2. Evidence on the Association between Risk Factors and the Level of Psychological Distress

First, we found that health and objective predictors played a substantial role in predicting the psychological distress level compared to sociodemographic predictors and perceived predictors. This finding was partially consistent with current studies of the link between built environment and body mass index [42,64], which had found that built environment predictors played a more essential role in affecting the related health outcomes than other predictors. Regarding the health predictors, we found that whether respondents had chronic disease and respondents’ self-rated health contributed over one third to predictive psychological distress. This finding was in line with the current COVID-19 study that a respondent with a chronic disease or with self-rated poor health had a significantly higher level of psychological distress [10,12,38]. This finding is plausible since an unpredicted virus outbreak could potentially exacerbate the negative effect on the level of psychological distress of respondents with a lower self-rated health level or chronic disease.

Regarding the objective predictors, an interesting finding of this study was that both distance to a park and distance to a COVID-19 hospital showed a non-linear relationship with psychological distress. Such a relationship fits an inverted V-shaped curve in general. This finding was in line with previous empirical studies suggesting that proximity to urban greenness had a significant inverted U-shape effect on health wellbeing [65]. A non-linear association has been found between AQI and the level of psychological distress. We observed a dramatic rise in the moderate level of psychological distress in regard to an AQI of between 40 and 50 and between 110 and 130, respectively. This result was consistent with a similar study, which indicated that the potential threshold effect of NO_2_ played a substantial role in health-based risk assessment [66]. One underlying mechanism might be that people are more sensitive to the dramatic increase in air pollutants that pose a threat to psychological health. In terms of the perceived predictors, an interesting finding was that perceived indoor air quality contributed 11.62% to predicting the level of psychological distress compared to AQI, which accounted for 6.64% when predicting the level of psychological distress. This finding indicates that perceived indoor air quality contributed more to predictive psychological distress compared to the ambient air pollution during the COVID-19 pandemic. One possible explanation might be that residents spent the majority of their time at home due to the lockdown restrictions during the very early stage of the pandemic. 

Regarding sociodemographic predictors, we found that respondents’ gender, age, marital status, household income, and geolocation did not affect the level of psychological distress significantly more than other risk factors, in addition to respondents’ neighborly relationships and educational attainment level. This finding partially challenges the common assumptions in the COVID-19 psychological health literature, as most studies have suggested that sociodemographic characteristics have played a significant role in influencing the level of psychological distress [12]. However, studies have not quantified which sociodemographic characteristics have played a more important role in affecting the level of psychological distress in addition to evidence indicating the significant or non-significant relationship between sociodemographic characteristics and the outcome. Overall, this study has provided a potential pathway to quantify the importance of different predictors on psychological health and has produced a more realistic association between related risk factors and psychological health by using the GBDT model. Further studies are advised to apply the GBDT to evaluate the direct and indirect effect of different noise exposures in both outdoor and perceived environments on mental health and to explore the potential preventive benefits of psychological noise attenuation by urban environment [67,68,69]. Additional research on exploring the potential pathway between green space exposure, air pollution, and psychological wellbeing is also recommended using GBDT [70,71,72,73].

### 4.3. Evidence from Gender Sensitive Analysis

Though we found that respondents’ gender did not have a significant effect on the level of psychological distress, we conducted additional gender-sensitive analysis to explore whether men and women would have different responses to psychological distress based on different risk factors. The results regarding the relative importance of different predictors among women were largely consistent with results in the total sample, while the results in men were partially in line with the results in the total sample. Specifically, for men, disease was considered the main risk factor that contributes to a higher level of psychological distress, while self-rated health contributes most to a higher level of psychological distress level among women. This finding indicates the robustness of the main findings and is in line with results in the total sample suggesting that health predictors contribute most to psychological distress for both men and women. Furthermore, we found that the influence of drinking on psychological distress was more pronounced for women than for men, which was consistent with previous studies indicating that the relationship between drinking and stress was more relevant to women than men [74,75]. Furthermore, in terms of the objective predictors, the results in men are largely consistent with the findings in the total sample. However, an interesting finding was that the distance to the nearest COVID-19 hospital contributed significantly to psychological distress in women. One possible explanation might be that women were more likely to perceive severe symptoms during the pandemic than men [76,77], and living close to the nearest COVID-19 hospital exacerbated such influences. Additional results show that men were more sensitive to the perceived environment but less sensitive to the impact of perceived indoor air quality on psychological distress, while women were more sensitive to the impact of perceived indoor air quality on psychological distress, which warrants further study.

### 4.4. Limitations

This study has several limitations. First, since our study applied a cross-sectional design, we cannot avoid the causal effect, which may lead to potential bias in the estimations. Nevertheless, our sample was collected at a relatively early stage of the outbreak of COVID-19 in China, which might minimize such bias to some extent. Second, this study used a non-random sampling design based on network invitation through WeChat, which might cause specific populations across the country to be under-represented in the sample. In our study, one underlying reason for the limited sample size of older adults might be that elderly people have limited access to mobile phones. Third, as perception variables were self-reported and variables were measured as the focal perception of the survey participants, which might also create potential bias. Lastly, empirical research might not control for the hypothesis; instead, an experimental method should be considered for future research if the necessary data are available. These limitations warrant future research to address these challenges.

## 5. Conclusions

Using individual items of data (*n* = 937) obtained from an online questionnaire between 20 January and 13 February, the early stage of the outbreak of coronavirus (COVID-19) in 2020, we quantified the relative importance of different risk factors in predicting the level of psychological distress by using the GBDT. The results from this study indicate that among all predictors, health predictors played the most important role in predicting the level of psychological distress. Though objective predictors contributed slightly more to predicting the level of psychological distress compared to perceived predictors, perceived indoor air quality played a more important role in predicting psychological distress compared to the ambient air pollution. This finding might be more significant during the COVID-19 pandemic, when respondents were compulsorily quarantined at home. Finally, we found that women and men respond differently to psychological distress based on different risk factors.

## Figures and Tables

**Table 1:** Definition and descriptive characteristics of variables conducted in this study.

Variables	Definition	*N*	Mean/%
Response Variable			
K6 Score (0–30) (mean (SD))	The Kessler Psychological Distress Scale, K6	937	9.3
Predictors			
Sex (*n*, %)	Female as the reference category		
	Male	325	65.3
	Female	612	34.7
Age (*n*, %)			
	18–24	172	18.4
	25–34	381	40.7
	35–44	238	25.4
	45–54	101	10.8
	55–64	36	3.8
	65–74	7	0.8
	75+	2	0.2
Marital Status (*n*, %)	Unmarried as the reference category		
	Married	570	60.8
	Unmarried	367	39.2
Education (*n*, %)			
	Illiteracy	29	3.1
	Primary	72	7.7
	Junior high school	181	19.3
	Technical secondary school	137	14.6
	High school	132	14.1
	College	156	16.7
	Undergraduate	169	18.0
	Master	49	5.2
	PhD and above	12	1.3
Household Income (*n*, %)			
	Monthly earnings of 3000 yuan	245	26.2
	Monthly earning 3000–10,000 yuan	368	39.3
	Monthly earning 10,000–20,000 yuan	183	19.5
	Monthly earning 20,000–30,000 yuan	69	7.4
	Monthly earning 30,000–50,000 yuan	29	3.1
	Monthly earning of 50,000 yuan	43	4.6
Smoke (*n*, %)			
	Current smoker	203	21.7
	Non-smoker	734	78.3
Drink (*n*, %)			
	Current drinker	188	20.1
	Non-current drinker	749	79.9
Physical exercise (*n*, %)			
	Never	211	22.5
	Physical activity only once per week	255	27.2
	Physical activity 2–4 times per week	295	31.5
	Physical activity 5–7 times per week	119	12.7
	Physical activity more than 7 times per week	57	6.1
Disease (*n*, %)			
	Have a chronic disease	178	81
	No chronic disease	759	19
Self-rated health (*n*, %)			
	Extremely poor health	35	3.7
	Poor health	97	10.4
	Neutral	360	38.4
	Good health	304	32.4
	Extremely good health	141	15.1
Neighborhood (*n*, %)			
	Extremely unsatisfied with the neighborly relationship	44	4.7
	Unsatisfied with the neighborly relationship	89	9.5
	Neutral	353	37.7
	Satisfied with the neighborly relationship	334	35.7
	Extremely satisfied with the neighborly relationship	117	12.5
Perception of the indoor air quality (*n*, %)			
	Extremely bad indoor air quality	41	4.4
	Bad indoor air quality	104	11.1
	Neutral	309	33.0
	Good indoor air quality	367	39.2
	Extremely good indoor air quality	16	12.3
Perception of overall environment quality (*n*, %)			
	Environments maintain in very poor quality	52	5.6
	Environments maintain in poor quality	123	13.1
	Neutral	405	43.2
	Environments maintain in good quality	263	28.1
	Environments maintain in very good quality	94	10.0
Perception of distance to the COVID-19 hospital			
	Very far, at least an hour’s drive	205	21.9
	Far, at least half hour’s drive	348	37.1
	Close, at least 10 min to 30 min drive	306	32.7
	Very close, 5 min drive	78	8.3
AQI (mean (SD))	Air quality index	937	81.1
Distance to the park (mean (SD), KM)	Direct distance from the residence to the nearest park	927	37.8
Distance to the hospital (mean (SD), KM)	Direct distance from the residence to the nearest hospital	937	67.1

**Table 2:** Importance of independent variables in predicting psychological distress K6 score.

Predictors	Relative Importance (%)	Rank
Health predictors	Total 42.32	
Disease	17.46	1
Self-rated health	16.23	2
Smoke	3.45	9
Drink	3.14	10
Physical exercise	2.04	13
Objective predictors	Total 23.49	
Distance to nearest parks	9.38	4
Distance to the nearest COVID-19 hospital	7.47	5
Air quality index (AQI)	6.64	7
Sociodemographic predictors	Total 17.91	
Neighbourly relationship	6.96	6
Education attainment level	4.37	8
Age	2.69	12
Marital status	1.45	15
Household Income	1.03	16
Urban	0.89	17
Gender	0.52	18
Perceived predictors	Total 16.26	
Perceived indoor air quality	11.62	3
Perceived distance to COVID-19 hospital	2.71	11
Perceived environment	1.93	14

**Table 3:** Importance of independent variables in predicting psychological distress K6 score between men and women.

Predictors	Men		Women	
	Relative Importance (%)	Rank	Relative Importance (%)	Rank
Health predictors	Total 31.31		Total 32.05	
Disease	16.87	1	3.18	9
Self -rated health	9.25	4	25.20	1
Smoke	1.56	12	0.75	15
Drink	0.63	17	0.80	14
Physical exercise	3.00	10	2.12	12
Objective predictors	Total 25.10		Total 32.01	
Distance to nearest parks	6.47	7	8.82	5
Distance to the nearest COVID-19 hospital	8.89	5	14.21	2
Air quality index (AQI)	9.74	3	8.98	4
Sociodemographic predictors	Total 27.57		Total 14.80	
Neighborly relationship	16.7	2	2.43	10
Education attainment level	5.05	9	4.65	7
Age	1.33	13	4.58	8
Marital status	0.97	16	0.28	17
Household Income	2.33	11	2.35	11
Urban	1.19	15	0.51	16
Perceived predictors	Total 18.93		Total 21.13	
Perceived indoor air quality	1.20	14	13.67	3
Perceived distance to COVID-19 hospital	5.98	8	1.25	13
Perceived environment	8.84	6	6.21	6

## Data Availability

Data sharing not applicable.

## References

[1] Li Q., Guan X., Wu P., Wang X., Zhou L., Tong Y., Ren R., Leung K.S., Lau E.H., Wong J.Y. (2020). Early transmission dynamics in Wuhan, China, of novel coronavirus–infected pneumonia. N. Engl. J. Med..

[2] Wang C., Horby P.W., Hayden F.G., Gao G.F. (2020). A novel coronavirus outbreak of global health concern. Lancet.

[3] Zhu N., Zhang D., Wang W., Li X., Yang B., Song J., Zhao X., Huang B., Shi W., Lu R. (2020). A novel coronavirus from patients with pneumonia in China, 2019. N. Engl. J. Med..

[4] Chen F., Zheng D., Liu J., Gong Y., Guan Z., Lou D. (2020). Depression and anxiety among adolescents during COVID-19: A cross-sectional study. Brain Behav. Immun..

[5] Vindegaard N., Benros M.E. (2020). COVID-19 pandemic and mental health consequences: Systematic review of the current evidence. Brain Behav. Immun..

[6] Cao W., Fang Z., Hou G., Han M., Xu X., Dong J., Zheng J. (2020). The psychological impact of the COVID-19 epidemic on college students in China. Psychiatry Res..

[7] Islam M.S., Ferdous M.Z., Potenza M.N. (2020). Panic and generalized anxiety during the COVID-19 pandemic among Bangladeshi people: An online pilot survey early in the outbreak. J. Affect. Disord..

[8] Moghanibashi-Mansourieh A. (2020). Assessing the anxiety level of Iranian general population during COVID-19 outbreak. Asian J. Psychiatry.

[9] Barzilay R., Moore T.M., Greenberg D.M., DiDomenico G.E., Brown L.A., White L.K., Gur R.C., Gur R.E. (2020). Resilience, COVID-19-related stress, anxiety and depression during the pandemic in a large population enriched for healthcare providers. Transl. Psychiatry.

[10] Chen Y., Jones C., Dunse N. (2021). Coronavirus disease 2019 (COVID-19) and psychological distress in China: Does neighbourhood matter?. Sci. Total Environ..

[11] Kujawa A., Green H., Compas B.E., Dickey L., Pegg S. (2020). Exposure to COVID-19 pandemic stress: Associations with depression and anxiety in emerging adults in the United States. Depress. Anxiety.

[12] Mazza C., Ricci E., Biondi S., Colasanti M., Ferracuti S., Napoli C., Roma P. (2020). A nationwide survey of psychological distress among Italian people during the COVID-19 pandemic: Immediate psychological responses and associated factors. Int. J. Environ. Res. Public Health.

[13] Qiu J., Shen B., Zhao M., Wang Z., Xie B., Xu Y. (2020). A nationwide survey of psychological distress among Chinese people in the COVID-19 epidemic: Implications and policy recommendations. Gen. Psychiatry.

[14] Joob B., Wiwanitkit V. (2020). Traumatization in medical staff helping with COVID-19 control. Brain Behav. Immun..

[15] Kang L., Ma S., Chen M., Yang J., Wang Y., Li R., Yao L., Bai H., Cai Z., Yang B.X. (2020). Impact on mental health and perceptions of psychological care among medical and nursing staff in Wuhan during the 2019 novel coronavirus disease outbreak: A cross-sectional study. Brain Behav. Immun..

[16] Song X., Fu W., Liu X., Luo Z., Wang R., Zhou N., Yan S., Lv C. (2020). Mental health status of medical staff in emergency departments during the Coronavirus disease 2019 epidemic in China. Brain Behav. Immun..

[17] Tan B.Y., Chew N.W., Lee G.K., Jing M., Goh Y., Yeo L.L., Zhang K., Chin H.-K., Ahmad A., Khan F.A. (2020). Psychological impact of the COVID-19 pandemic on health care workers in Singapore. Ann. Intern. Med..

[18] Husky M.M., Kovess-Masfety V., Swendsen J.D. (2020). Stress and anxiety among university students in France during Covid-19 mandatory confinement. Compr. Psychiatry.

[19] Martínez-Lorca M., Martínez-Lorca A., Criado-Álvarez J.J., Armesilla M.D.C. (2020). The fear of COVID-19 scale: Validation in Spanish university students. Psychiatry Res..

[20] Kong X., Zheng K., Tang M., Kong F., Zhou J., Diao L., Wu S., Jiao P., Su T., Dong Y. (2020). Prevalence and factors associated with depression and anxiety of hospitalized patients with COVID-19. MedRxiv.

[21] Muruganandam P., Neelamegam S., Menon V., Alexander J., Chaturvedi S.K. (2020). COVID-19 and Severe Mental Illness: Impact on patients and its relation with their awareness about COVID-19. Psychiatry Res..

[22] Wang Y., Duan Z., Ma Z., Mao Y., Li X., Wilson A., Qin H., Ou J., Peng K., Zhou F. (2020). Epidemiology of mental health problems among patients with cancer during COVID-19 pandemic. Transl. Psychiatry.

[23] Mosley S. (2014). Environmental history of air pollution and protection. The Basic Environmental History.

[24] Brunekreef B., Holgate S.T. (2002). Air pollution and health. Lancet.

[25] Kioumourtzoglou M.-A., Power M.C., Hart J.E., Okereke O.I., Coull B.A., Laden F., Weisskopf M.G. (2017). The association between air pollution and onset of depression among middle-aged and older women. Am. J. Epidemiol..

[26] Power M.C., Kioumourtzoglou M.-A., Hart J.E., Okereke O.I., Laden F., Weisskopf M.G. (2015). The relation between past exposure to fine particulate air pollution and prevalent anxiety: Observational cohort study. BMJ.

[27] Sass V., Kravitz-Wirtz N., Karceski S.M., Hajat A., Crowder K., Takeuchi D. (2017). The effects of air pollution on individual psychological distress. Health Place.

[28] Pinault L., Thomson E.M., Christidis T., Colman I., Tjepkema M., van Donkelaar A., Martin R.V., Hystad P., Shin H., Crouse D.L. (2020). The association between ambient air pollution concentrations and psychological distress. Health Rep..

[29] He G., Chen Y., Wang S., Dong Y., Ju G., Chen B. (2020). The Association between PM_2.5_ and Depression in China. Dose-Response.

[30] Zijlema W.L., Wolf K., Emeny R., Ladwig K.-H., Peters A., Kongsgård H., Hveem K., Kvaløy K., Yli-Tuomi T., Partonen T. (2016). The association of air pollution and depressed mood in 70,928 individuals from four European cohorts. Int. J. Hyg. Environ. Health.

[31] WorsePolluted Indoor Air Pollution. https://www.worstpolluted.org/projects_reports/display/59.

[32] EPA An Introduction to Indoor Air Quality. https://www.epa.gov/indoor-air-quality-iaq/introduction-indoor-air-quality.

[33] Kim S., Senick J.A., Mainelis G. (2019). Sensing the invisible: Understanding the perception of indoor air quality among children in low-income families. Int. J. Child Comput. Interact..

[34] Dunleavy G., Bajpai R., Tonon A.C., Cheung K.L., Thach T.-Q., Rykov Y., Soh C.-K., de Vries H., Car J., Christopoulos G. (2020). Prevalence of psychological distress and its association with perceived indoor environmental quality and workplace factors in under and aboveground workplaces. Build. Environ..

[35] Chen H., Liu Y., Zhu Z., Li Z. (2017). Does where you live matter to your health? Investigating factors that influence the self-rated health of urban and rural Chinese residents: Evidence drawn from Chinese general social survey data. Health Qual. Life Outcomes.

[36] Sønderskov K.M., Dinesen P.T., Santini Z.I., Østergaard S.D. (2020). The depressive state of Denmark during the COVID-19 pandemic. Acta Neuropsychiatr..

[37] Wang H., Xia Q., Xiong Z., Li Z., Xiang W., Yuan Y., Liu Y., Li Z. (2020). The psychological distress and coping styles in the early stages of the 2019 coronavirus disease (COVID-19) epidemic in the general mainland Chinese population: A web-based survey. PLoS ONE.

[38] Wang C., Pan R., Wan X., Tan Y., Xu L., Ho C.S., Ho R.C. (2020). Immediate psychological responses and associated factors during the initial stage of the 2019 coronavirus disease (COVID-19) epidemic among the general population in China. Int. J. Environ. Res. Public Health.

[39] Rahman M.A., Hoque N., Alif S.M., Salehin M., Islam S.M.S., Banik B., Sharif A., Nazim N.B., Sultana F., Cross W. (2020). Factors associated with psychological distress, fear and coping strategies during the COVID-19 pandemic in Australia. Glob. Health.

[40] Sfendla A., Hadrya F. (2020). Factors associated with psychological distress and physical activity during the COVID-19 pandemic. Health Secur..

[41] Ding C., Wang D., Ma X., Li H. (2016). Predicting short-term subway ridership and prioritizing its influential factors using gradient boosting decision trees. Sustainability.

[42] Yin C., Cao J., Sun B. (2020). Examining non-linear associations between population density and waist-hip ratio: An application of gradient boosting decision trees. Cities.

[43] Hastie T., Tibshirani R., Friedman J. (2009). The Elements of Statistical Learning: Data Mining, Inference, and Prediction.

[44] Ma X., Ding C., Luan S., Wang Y., Wang Y. (2017). Prioritizing influential factors for freeway incident clearance time prediction using the gradient boosting decision trees method. IEEE Trans. Intell. Transp. Syst..

[45] Friedman J.H. (2001). Greedy function approximation: A gradient boosting machine. Ann. Stat..

[46] Schonlau M. (2005). Boosted regression (boosting): An introductory tutorial and a Stata plugin. Stata J..

[47] Breiman L., Friedman J., Stone C.J., Olshen R.A. (1984). Classification and Regression Trees.

[48] Kessler R.C., Andrews G., Colpe L.J., Hiripi E., Mroczek D.K., Normand S.L.T., Walters E.E., Zaslavsky A.M. (2002). Short screening scales to monitor population prevalences and trends in non-specific psychological distress. Psychol. Med..

[49] Fushimi M., Saito S., Shimizu T., Kudo Y., Seki M., Murata K. (2012). Prevalence of psychological distress, as measured by the Kessler 6 (K6), and related factors in Japanese employees. Community Ment. Health J..

[50] Hill T.D., Burdette A.M., Hale L. (2009). Neighborhood disorder, sleep quality, and psychological distress: Testing a model of structural amplification. Health Place.

[51] Ugolini F., Massetti L., Pearlmutter D., Sanesi G. (2021). Usage of urban green space and related feelings of deprivation during the COVID-19 lockdown: Lessons learned from an Italian case study. Land Use Policy.

[52] Zhu Y., Cao L., Xie J., Yu Y., Chen A., Huang F. (2020). Using social media data to assess the impact of COVID-19 on mental health in China. Psychological Medicine.

[53] Breslau N., Roth T., Rosenthal L., Andreski P. (1996). Sleep disturbance and psychiatric disorders: A longitudinal epidemiological study of young adults. Biol. Psychiatry.

[54] Lawrence D., Mitrou F., Zubrick S.R. (2011). Non-specific psychological distress, smoking status and smoking cessation: United States National Health Interview Survey 2005. BMC Public Health.

[55] Marchand A., Demers A., Durand P., Simard M. (2003). Occupational variations in drinking and psychological distress: A multilevel analysis. Work.

[56] Meerlo P., Sgoifo A., Suchecki D. (2008). Restricted and disrupted sleep: Effects on autonomic function, neuroendocrine stress systems and stress responsivity. Sleep Med. Rev..

[57] Turner J., Kelly B. (2000). Emotional dimensions of chronic disease. West. J. Med..

[58] Weyerer S., Kupfer B. (1994). Physical exercise and psychological health. Sports Med..

[59] Cronbach L.J. (1951). Coefficient alpha and the internal structure of tests. Psychometrika.

[60] Nunnally J.C. (1994). Psychometric Theory 3E.

[61] George D. (2011). SPSS for Windows Step by Step: A Simple Study Guide and Reference, 17.0 Update, 10/e.

[62] Bolarinwa O.A. (2015). Principles and methods of validity and reliability testing of questionnaires used in social and health science researches. Niger. Postgrad. Med. J..

[63] Greenwell B., Boehmke B., Cunningham J., GBM D. (2019). gbm: Generalized Boosted Regression Models. R Package Version 2.1. 5. https://cran.r-project.org/package=gbm.

[64] Wang X., Shao C., Yin C., Guan L. (2021). Disentangling the comparative roles of multilevel built environment on body mass index: Evidence from China. Cities.

[65] Ma B., Zhou T., Lei S., Wen Y., Htun T.T. (2019). Effects of urban green spaces on residents’ well-being. Environ. Dev. Sustain..

[66] Zhao S., Liu S., Sun Y., Liu Y., Beazley R., Hou X. (2020). Assessing NO_2_-related health effects by non-linear and linear methods on a national level. Sci. Total Environ..

[67] Dzhambov A.M., Dimitrova D.D. (2014). Urban green spaces’ effectiveness as a psychological buffer for the negative health impact of noise pollution: A systematic review. Noise Health.

[68] Ma J., Li C., Kwan M.-P., Kou L., Chai Y. (2020). Assessing personal noise exposure and its relationship with mental health in Beijing based on individuals’ space-time behavior. Environ. Int..

[69] Yang M., Dijst M., Faber J., Helbich M. (2020). Using structural equation modeling to examine pathways between perceived residential green space and mental health among internal migrants in China. Environ. Res..

[70] Liu Y., Wang R., Grekousis G., Liu Y., Yuan Y., Li Z. (2019). Neighbourhood greenness and mental wellbeing in Guangzhou, China: What are the pathways?. Landsc. Urban Plan..

[71] Liu Y., Wang R., Xiao Y., Huang B., Chen H., Li Z. (2019). Exploring the linkage between greenness exposure and depression among Chinese people: Mediating roles of physical activity, stress and social cohesion and moderating role of urbanicity. Health Place.

[72] Wang R., Yang B., Yao Y., Bloom M.S., Feng Z., Yuan Y., Zhang J., Liu P., Wu W., Lu Y. (2020). Residential greenness, air pollution and psychological well-being among urban residents in Guangzhou, China. Sci. Total Environ..

[73] Wang R., Liu Y., Xue D., Yao Y., Liu P., Helbich M. (2019). Cross-sectional associations between long-term exposure to particulate matter and depression in China: The mediating effects of sunlight, physical activity, and neighborly reciprocity. J. Affect. Disord..

[74] Kuntsche E., Wicki M., Windlin B., Roberts C., Gabhainn S.N., van der Sluijs W., Aasvee K., de Matos M.G., Dankulincová Z., Hublet A. (2015). Drinking motives mediate cultural differences but not gender differences in adolescent alcohol use. J. Adolesc. Health.

[75] Rodriguez L.M., Litt D.M., Stewart S.H. (2020). Drinking to cope with the pandemic: The unique associations of COVID-19-related perceived threat and psychological distress to drinking behaviors in American men and women. Addict. Behav..

[76] Wang Q., Liu H., Ren Z., Xiong W., He M., Fan X., Guo X., Li X., Shi H., Zha S. (2020). Gender difference in the association of coping styles and social support with psychological distress among patients with end-stage renal disease. PeerJ.

[77] Shechter A., Diaz F., Moise N., Anstey D.E., Ye S., Agarwal S., Birk J.L., Brodie D., Cannone D.E., Chang B. (2020). Psychological distress, coping behaviors, and preferences for support among New York healthcare workers during the COVID-19 pandemic. Gen. Hosp. Psychiatry.

